# Association of homocysteine with body mass index, blood pressure, HbA1c and duration of diabetes in type 2 diabetics

**DOI:** 10.12669/pjms.346.16032

**Published:** 2018

**Authors:** Zulfania, Adnan Khan, Sohaib Rehman, Tahir Ghaffar

**Affiliations:** 1 *Dr. Zulfania, M.Phil. Department of Physiology, Rehman Medical College, Peshawar, Pakistan*; 2 *Dr. Adnan Khan, MBBS. Postgraduate Resident (PGR 1) Paediatrics, Rehman Medical Institute, Peshawar, Pakistan*; 3 *Dr. Sohaib Rehman, MPhil. Department of Biochemistry, Rehman Medical College, Peshawar, Pakistan*; 4 *Tahir Ghaffar, FCPS. Department of Endocrinology, Lady Reading Hospital, Peshawar, Pakistan*

**Keywords:** Homocysteine, BMI, Glycated hemoglobin, Diabetes mellitus

## Abstract

**Objective:**

To determine the Homocysteine levels in type 2 diabetics and correlate homocysteine with HbA1c levels, BMI, blood pressure and duration of diabetes.

**Methods:**

This cross-sectional study was conducted in Endocrinology Unit of Hayatabad Medical Complex (HMC) Peshawar and Rehman Medical Institute (RMI) Peshawar over a period of six months from July 2015 to December 2015. Data was recorded and analyzed in SPSS v 20. P value of less than 0.05 was taken as significant. Bivariate Pearson’s correlation test was used to see the relationship between homocysteine and BMI, systolic BP and duration of diabetes.

**Results:**

One hundred twenty five patients were included in our study in which female were 68% and 32% were male with mean age of 51.45 ±8.37 years. Mean BMI expressed in kg/m^2^ was 28.71±4.76, mean systolic blood pressure was 130±20.98 mmHg, mean diastolic blood pressure was 83.36±11.28 mmHg and mean duration of diabetes was 7.018± 6.18 years. Significant correlation was found between systolic blood pressure (r: 0.239, p: 0.007) and duration of diabetes with homocysteine (r: 0.302, p: 0.001). The correlation of homocysteine with HbA1c and BMI was not significant.

**Conclusion:**

Systolic blood pressure and duration of diabetes showed a significant positive correlation with homocysteine. The correlation of homocysteine with HbA1c was not certain from researcher’s point of view and further studies of larger sample size and longer duration must be conducted to ascertain the association between the two variables.

## INTRODUCTION

Diabetes is a metabolic disease characterized by hyperglycemia due to defective secretion of insulin, its action, or both.^1^ WHO has reported that 30 million people were suffering from diabetes mainly type II, worldwide in 1985; the number increased to 217 million in 2005 and is expected to touch the figure of 366 million by the year 2030.^2^ WHO survey in 1995 showed that Pakistan was at the 8^th^ position in top ten countries having high diabetic prevalence. The same survey has estimated that in the year 2025, Pakistan will be on the 4^th^ position with 14.5 million people having diabetes.^3^ About 7.2 million people were suffering from diabetes in 2012.^4^ The prevalence of diabetes has reached to 7.89% in Pakistan in 2015.^5^ In other countries of South East Asia the prevalence of diabetes varied much in 2014, with Mauritius having a prevalence of 14.8%, India had 9.1%, Sri Lanka 7.6%, Bangladesh 6.3%, Bhutan 5.8%, Nepal 4.9% and with Maldives 4.8%.^6^

Homocysteine (Hcy), formed after methionine demethylation, is a sulfur containing amino acid. Methionine being an essential amino acid is obtained from recycling it or from diet.^7^ Hyperhomocysteinemia (HHcy) is a well-known risk factor of atherosclerosis.^8^,^11^ It can promote all the factors which can lead to initiation of process of atherosclerosis including proliferation of vascular wall smooth muscle, and increasing oxidative stress.^7^,^12^

A number of studies have shown a positive association of coronary artery disease, stroke and venous thrombosis with increased plasma Hcy levels.^13^,^14^ Boushey *et al*. showed that increasing plasma Hcy levels 5 μmol/L above normal increases the cardiovascular risk to 1.6-1.8 times.^13^ A Meta-analysis done in 2005 showed that 25% increase in total Hcy is associated with a 10% increased risk of cardiovascular events and 20% advanced risk of stroke.^14^ The mechanisms involved in endothelial dysfunction caused by HHcy include inhibition of nitric oxide, endothelium- derived hyperpolarizing factor, regulation of prostanoids, activation of angiotensin II receptor 1, oxidative stress and endothelin-1 activation.^15^

Literature from this part of the world appears scarce. No comprehensive study has been conducted in the recent past. Thus, the aim of this study was to determine the Hcy levels in type 2 diabetics and correlate Hcy with HbA1c levels, BMI, blood pressure and duration of diabetes. The results of this study will provide us with local statistics of the homocysteine as a risk factor in cardiovascular disease in type II diabetes and this will open window for further research.

## METHODS

This cross sectional study was conducted in Endocrinology Unit of Hayatabad Medical Complex (HMC) Peshawar and Rehman Medical Institute (RMI), Peshawar over a period of six months from July 2015 to December 2015. The study includes diagnosed cases of Type 2 diabetes, both male and female, aged 40-60 years old without any complications. All patients who had type 1 diabetes mellitus and Patients with anemia or taking vitamin B supplements, pregnant or breastfeeding women were excluded. One hundred twenty five patients were recruited in our study by taking 37%^16^ prevalence of increased Hcy in type II diabetes.

### Data Collection Procedure

Data about demography medical history and general physical examination was recorded. Body mass index was calculated by taking height and weight. The auscultatory method of Blood Pressure (B.P) measurement was used and B.P taken in rested sitting position by mercury sphygmomanometer and Littman stethoscope. Both systolic and diastolic B.P was recorded. A 3ml fasting blood sample was taken from the patients under aseptic conditions using disposable syringe. The whole blood was used to measure HbA1c through immunoassay method and then centrifuged in *Beckman Allegra TM 6R* centrifuge at 3000 revolutions per minutes for 10 minutes to obtain plasma. The plasma was separated in eppendorff tubes and froze at -80°C for analysis of tHcy. Hcy was measured through Chemiluminescentmicroparticle Immunoassay (CMIA)

### Data Analysis

Data was recorded and analyzed in SPSS v 20. Mean + SD was calculated for numerical variables like age, BMI, serum Hcy level, HbA1C levels systolic blood pressure and duration of diabetes. Frequencies & percentages was calculated for categorical variables like gender’s p value of less than 0.05 was taken as significant. Bivariate Pearson’s correlation test was used to see the relationship between Hcy and |BMI, systolic BP and duration of diabetes.

## RESULTS

One hundred twenty five patients were included in our study in which female were 85(68%) and 40(32%) were male with mean age of 51.45 ± 8.37 years. Mean BMI expressed in kg/m^2^ was 28.71±4.76 20(16%) had normal BMI, 60(48%) were overweight and 45(36%) were obese. Out of 20 patients having normal BMI 12(60%) were females and 8(40%) were males, among 60 overweight patients 35(58.33%) were females and 25(41.66%) were males and altogether in 45 obese 38 were females and seven were males. Good glycemic control is indicated by HbA1c0 ≤7% and poor glycemic control is indicated by HbA1c0 > 7%. five male patients (4%) have good glycemic control while 35 male patients (28%) have poor glycemic control. The data in the given table also indicates that 11 females (8.8%) have good glycemic control and 74 (59.2%) have poor glycemic control. These two groups were not significantly different from each other (p= 0.945).

**Table-I:** Demography and vital signs.

Sr. No	Variables	Mean	Standard Deviation
1	Age (years)	51.45	8.37
2	BMI (kg/m^2^)	28.71	4.76
3	Systolic blood pressure (mmHg)	130	20.98
4	Diastolic blood pressure (mmHg)	83.36	11.28
5	Duration of diabetes (years)	7.018	6.18

Mean BMI expressed in kg/m^2^ was 28.71±4.76, mean systolic blood pressure was 130±20.98 mmHg, mean diastolic blood pressure was 83.36±11.28 mmHg and mean duration of diabetes was 7.018± 6.18 years.

There was significant correlation of systolic blood pressure and duration of diabetes with Hcy. However, the correlation of Hcy with HbA1c and BMI was not significant.

**Fig.1:**
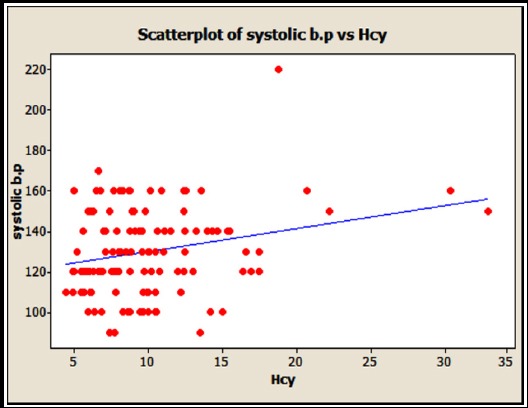
Correlation of Hcy and systolic blood pressure (with r= 0.239 and p = 0.007).

Fig.1 shows significant positive correlation of Hcy and systolic blood pressure (with r= 0.239 and p = 0.007).

**Fig.2:**
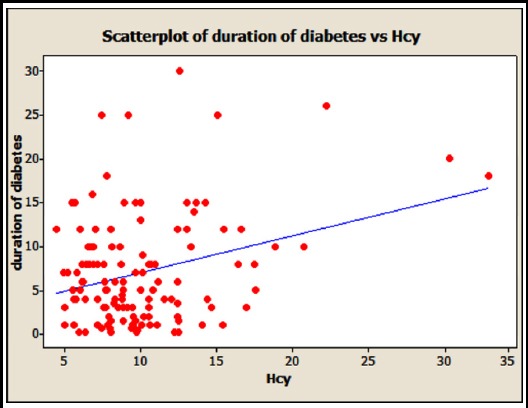
Correlation of Hcy duration of diabetes and Hcy (r= 0.302 and P-Value = 0.001)

Ssignificant positive correlation of duration of diabetes and Hcy (0.302 P-Value = 0.001) is shown in Fig-2.

## DISCUSSION

The present study revealed no correlation of Hcy and HbA1c. These findings endorse the report of other studies including a study conducted in Iran in which healthy controls were compared with type 2 diabetics having either good or poor glycemic control. They observed that although Hcy levels were higher in diabetics but not significantly different among various groups. They concluded that glycemic control does not influence Hcy levels and no correlation exists between the two variables.^17^ Other researchers like Hoogeven et al. also found no association of glycemic control and Hcy.^18^ A study in which Aghamohammadi et al. studied the correlation of Hcy and HbA1c in 70 type 2 diabetic males reported no statistically significant association between the two variables.^19^ This study also had the same conclusion that just keeping glycemic control is not sufficient for maintaining lower Hcy levels but other measures such as use of vitamin B12 and folic acid is also necessary for diabetics. The effect of improved glycemic control and insulin sensitivity on Hcy levels was investigated by Pouwels et al. and they also confirmed that HbA1c levels have no influence on Hcy.^20^ A study conducted on Kenyan type 2 diabetics without any cardiovascular disease also showed no effect of HbA1c levels on Hcy.^21^

**Table-II:** Pearson correlation of variables with Hcy.

Variables	Hcy correlation (r)	p- value
HbA1c	-0.052	0.576
systolic blood pressure	0.239	0.007*
Duration of diabetes	0.302	0.001*
BMI	-0.101	0.261

No correlation of Hcy with BMI was found in the present study. Similar findings were reported by a study conducted on Kenyan type 2 diabetics, which showed no correlation of BMI with Hcy levels in patients without cardiovascular disease.^21^ Another study of type two diabetics without cardiovascular disease also showed no association of BMI and Hcy.^22^ However a case control study conducted in Gaza where association of Hcy and BMI in type 2 diabetics having diabetic complications were compared with healthy volunteers, the correlation was reported as significantly positive.^23^ Hence it may be concluded that type 2 diabetics without micro and macrovascular complications do not show any correlation between Hcy and BMI. Furthermore the Hcy and lipid disorders may coexist, but as independent risk factors.

Systolic B.P showed a significant positive correlation with Hcy in the present study, which is in agreement with the finding of Passaro et al.^24^ However there was no significant correlation between the two variables in study conducted in Kenyan type 2 diabetics.^21^

The present study showed a significant positive correlation of duration of diabetes and Hcy levels. Similar findings were observed in a study by Sonkar et al in which plasma Hcy levels were correlated with duration and complications of type 2diabetes.^25^ A study conducted on patients with diabetic retinopathy also showed a significant positive correlation of duration of diabetes with Hcy.^26^ Similar findings were observed in another study conducted to assess plasma Hcy levels in type 2 diabetics which showed that Hcy levels increased with increasing duration of diabetes.^27^ Therefore we suggest that increased duration of diabetes increases Hcy levels which may be due to decreased vitamin B12 levels in such patients.

## CONCLUSION

It can be inferred that systolic blood pressure and duration of diabetes showed a significant positive correlation with Hcy. The correlation of Hcy with HbA1c was not certain from researcher’s point of view and further studies of larger sample size and longer duration must be conducted to ascertain the association between the two variables.

### Authors’ Contribution

**Z, AK:** Conceived, designed and did statistical analysis & editing of manuscript.

**SR, TG, AK, Z:** Did data collection and manuscript writing.

**AK:** Did review and final approval of manuscript.
